# The Endless Grief in Waiting: A Qualitative Study of the Relationship between Ambiguous Loss and Anticipatory Mourning amongst the Relatives of Missing Persons in Italy

**DOI:** 10.3390/bs10070110

**Published:** 2020-07-06

**Authors:** Ines Testoni, Chiara Franco, Lorenza Palazzo, Erika Iacona, Adriano Zamperini, Michael Alexander Wieser

**Affiliations:** 1 Department of Philosophy, Sociology, Pedagogy and Applied Psychology, University of Padova, 35131 Padova, Italy; ines.testoni@unipd.it (I.T.); chiara.franco25@gmail.com (C.F.); lorenza.palazzo92@gmail.com (L.P.); erika.iacona@unipd.it (E.I.); adriano.zamperini@unipd.it (A.Z.); 2 Arts Therapies Research Center, University of Haifa, Haifa 3498838, Israel; 3 Institute of Psychology, University of Klagenfurt, 9020 Klagenfurt am Wörthersee, Austria

**Keywords:** missing people, disappearance, grief, anticipatory mourning, ambiguous loss, waiting

## Abstract

This article presents the results of a qualitative study aiming to consider the relationship between ambiguous loss and anticipatory mourning amongst relatives of missing people in Italy. Eight people participated in the research, narrating their experiences of losing a beloved person (one found alive, three found dead, and four still missing). Findings suggest the presence of a particular form of ambiguous loss, characterised by traits typical of both prolonged and traumatic grief. These findings describe how families are faced with an emotional vortex related to a never-ending wait, and how the mourning is solved only when the missing person is found dead or alive. The discovery of a corpse is traumatic but it allows mourners to fully recognise their grief. When a person is found, it changes the relationship in a positive way. When neither of these events happen, mourners have two different kinds of reactions: they experience either a prolonged grief or a drive to solve their suffering by helping other people (post-traumatic growth). In this study, it is highlighted how a community can be useful or detrimental in this process, and the importance of psychological and social support to prevent significant clinical outcomes is stressed.

## 1. Introduction

The loss of a beloved person, whether due to death or separation, always causes grief. While the literature on mourning caused by death is now very extensive, there are few studies on mourners suffering because their loved one is missing. For human beings, grief is a natural reaction to loss, though its symptoms vary across individuals. Some exhibit resilient reactions to loss with little psychological distress, whereas others exhibit grief reactions with clinically significant symptoms of depression, anxiety, posttraumatic stress disorder (PTSD) and complicated grief [1,2]. In addition, the experience of loss varies depending on the circumstances of the death. Unnatural, violent and sudden deaths entail a higher risk of psychopathological symptoms [2,3]. The disappearance of a beloved person can be a particularly stressful form of loss. Scholars have observed that grief reactions following a disappearance are a normal response to an abnormal situation [4,5]. If the type of grief experienced by a bereaved family reflects the circumstances of their loss, then an unresolved grief may be the result of an unresolved loss [6,7].

Pauline Boss defined the typical form of unresolved loss as ‘ambiguous loss’ [8], and she described two basic types depending on the manner of disappearance. In the first type, the missing person is physically absent (or ‘physically missing’) but psychologically present because their relatives are preoccupied with the disappeared and cannot determine whether they are dead or alive. In the second type, the ‘missing’ person is physically present but perceived as psychologically absent (or ‘psychologically missing’)—that is, emotionally and cognitively unavailable to those around them—because of depression, addiction, dementia, or the sudden appearance of other severe mental illnesses that cause prolonged disorders of consciousness [3,4,5,6,7,8]. Boss is particularly interested in the effect of these forms of loss on the families left behind, who are commonly concerned with finding coping strategies to deal with the absence of their beloved. Many family members experience relationship conflicts, somatisation and feelings of helplessness, depression and anxiety. These negative effects occur for several reasons: ambiguity is confusing, people cannot make cognitive sense of the situation, and not knowing whether the family member will return prevents the reconstruction of family and marital roles, rules and rituals. Ambiguity destroys the customary markers of life or death, so a person’s distress is never validated [9]. The community loses patience with the lack of closure, and families become isolated. Ambiguity causes even the strongest of people to question their view of the world as a fair, safe and understandable place. Finally, ambiguous loss that persists for a long time is physically and emotionally exhausting [4,5,6,7,8,9,10]. With regards to the first type of loss, Boss’s research considered the cases of soldiers declared missing, relatives who disappeared during civil conflicts—such as kidnapped people (‘desaparecido’, disappeared) in Argentina, Brazil, Columbia, Chile, Panama, Peru, Mexico and other countries—and individuals unaccounted for after terrorist attacks, such as ethnic cleansing in Europe (Kosovo) and Africa (Rwanda) or the September 11 attacks in the US [5,10]. In the present study, we consider the kind of mourning provoked by the ambiguous loss of individuals without an apparent cause (that is, in the absence of war, disaster, conflicts and so on).

In Italy, the Codice Civile (Civil Code, art 48) indicated that a person’s disappearance consists of their withdrawal from home without giving information about their fate. The Decree of the President of the Republic (DPR 31 July 2007) introduced the figure of the Extraordinary Commissioner for Missing Persons, who draws up reports containing a census of missing persons from the previous six months. From 1974 to 2019 [11], the total number of missing persons in Italy was 245,012; the total number of those traced was 183,976; and the total number of those who still need to be traced was 61,036. The main causes of disappearance are: voluntary removal; removal of children from institutions/communities; psychological disorders; child abduction by a parent or other family member; and crime.

In Italy, the attention given to this problem is due to the growing interest of media, social networks, and associations aimed at raising public awareness and improving searches for missing people. For example, the television program *Chi ha visto l’ha visto?* (*Who Saw Him/Her*), which aired from 1989 to 2006, solved 236 cases thanks to the participation of viewers (65% of total reported missing persons) [12]. These data highlight the importance of community participation in finding the missing person or their corpse. When neither of those events happen, a long search for information begins for the missing person’s family members. The type of grief experienced is similar to that of ambiguous loss [4,8,9], which is characterised by the absence of a corpse that permits definitive separation [6].

Because ambiguous loss implies an unconfirmed loss, it defies closure and prevents the resolution of grief [3,6]. In fact, the primary aspect that distinguishes ambiguous loss from clear-cut loss is what Boss initially called ‘boundary ambiguity’, or the difficulty of establishing the boundary between ‘who is in or out of a particular family’ [5], p. 553. Both types of ambiguous loss identified by Boss require the family to reconstruct their family system within the paradox of absence–presence [10]. Boundary ambiguity can vary in intensity, and it can threaten individual and relational well-being, preventing stress management [5].

Furthermore, disappearance is a relational loss [6]. The definition of ‘missing person’ assumes the existence of someone who misses the disappeared. To further investigate the phenomenon, it is therefore relevant to understand the meaning of ambiguous loss and the way that it involves the relatives of a missing person [13]. Indeed, even though there are numerous cases of missing persons, there is still scant literature about ambiguous loss related to missing persons, as well as a lack of attention to those ‘people left behind’ and the ways in which they manage these never-ending losses [14].

The phenomenon of missing persons was first studied by Eric Lindemann in the 1940s. He coined the term ‘Anticipatory Grief’ [15], which was introduced into the literature with the term ‘Anticipatory Mourning’, first by Elisabeth Kübler-Ross [16] and then by Therese Rando [17]. Rando’s studies on ‘anticipatory mourning’ and ‘anticipatory grief’ triggered wide discussions [18], however, for the purpose of our discussion, the second term subsumes the first and describes the ambiguous condition of perceiving someone who is still alive as already dead, causing the loved ones to suffer the loss in advance. In Rando’s opinion, anticipatory mourning encompasses seven operations: grief and mourning, coping, interaction, psychosocial reorganization, planning, balancing conflicting demands and facilitating an appropriate death. Such operations characterise a psychological context of adaptational demands, which is caused by experiences of loss and trauma following the awareness that death is near at hand [17]. Indeed, Rando integrated the two definitions following the precedent of Lindemann’s epigones [19,20,21], who studied parents’ responses to their child’s terminal illness. The concept of anticipatory grief/mourning is used both to indicate emotional reactions prior to exitus and those related to a separation not necessarily determined by death.

Before Rando, anticipatory grief/mourning had been examined by Elisabeth Kübler-Ross [16], who described the denial, anger, bargaining, depression, acceptance (DABDA) model of coping with death. This model is comprised of five stages: denial, anger, bargaining, depression and acceptance. According to Kübler-Ross and other scholars, this process unfolds in generally the same way regardless of whether the death has already occurred. [22,23]. Hope holds a prominent position in Kübler-Ross’s five-phase model because it forms the background to the continuous variation in emotional experiences arising from anticipated loss. Even in the literature on ambiguous loss, hope occupies a fundamental role. The path of hope is not linear, but incessant and flexible: initially, it is based on the idea that the beloved person is located in a safe place; progressively, it acknowledges the condition of disappearance and focuses on achieving closure; concurrently, hope enables those ‘left behind’ to achieve a peaceful state despite the situation [14,24]. Those left behind need hope because it allows them to ascribe meaning to their loss and, in some circumstances, to maintain a positive connection with the missing person [6,24,25,26]. The main difference between the academic studies of ambiguous loss and anticipatory grief/mourning is that in the first case, attention is primarily paid to family dynamics, whereas in the second case, attention is paid to individual experiences of suffering and bargaining with the phantom presence of death. In both cases, death is simultaneously present and absent, and the most important difficulty is managing this haziness.

This phenomenon has not yet been studied in Italy. Based on these foundational models, we endeavoured to carry out grounded qualitative research to find out which aspects among those described by Boss, Kübler-Ross and Rando are most important. The study of the effects of this form of grieving may be useful when organizing suitable community psychological support interventions.

Given the lack of research in this specific field and the opportunity to consider how to help people suffering from this type of loss, we realised the study to better understand the relationship between the different forms in which the concept of anticipatory grief/mourning has been represented, and the ambiguous loss.

## 2. Materials and Methods

### The Qualitative Study

The study followed the American Psychological Association’s Ethical Principles and Code of Conduct and the principles of the Declaration of Helsinki; furthermore, it was approved by the Ethics Committee of the University of Padua (Prot. 2868 n. 5A06A9EF69B8970084C83EDB4E2DD3FD). Participants were informed about the study’s aims and procedures and they were assured that participation was voluntary and that their responses would remain confidential. Informed consent was obtained from all participants.

This study utilised the Grounded Theory perspective and involved qualitative research in psychology with in-depth interviews, because this approach makes it easier to understand subjective stories [27,28,29]. The Grounded Theory offers a practical and flexible approach to interpret complex social phenomena. This qualitative method does not start with testing an existing hypothesis, but uses the empirical data to generate concepts and theories. Thus, one of the benefits of this perspective is the actively reflective position of the researcher while interacting with data, which involves the generation of analytical categories and the identification of relationships between them [30,31,32]. The thematic analysis method was adopted, because it offers an accessible and theoretically flexible approach and it is consistent with the framework of the Grounded Theory [30].

The primary aim was to trace the relationships between ambiguous loss and anticipatory mourning as experienced by people who have dealt with the disappearance of a loved one. We also wanted to understand how these grievers experienced community support. Eight participants (six females and two males; average age: 50; SD 10) were involved thanks to the support of an association dedicated to finding missing persons. The sampling method was not random because we preferred to recruit individuals who were motivated to participate in the project. All the participants used various instruments to search for loved ones, and six participants had asked for help from the *Chi l’ha visto* TV program through a public appeal reserved for the search of missing persons. One missing person was found alive, three were found dead, and four have not yet been found. The degree of relatedness was: four parents, three children, and one aunt (Table 1). The duration of the disappearances varied from 10 days to 26 years. Before the interviews began, the topics and the focus of the study were explained, and then the participants were asked to sign the informed consent form. The interviews were conducted between June and July 2019 with a mean duration of 40 min. The names used in this text are fictional.

The interviewer followed an elastic grid of topics to support the dialogues, and focused on the following: biographical narrations about the missing loved ones and how they disappeared; emotions related to the wait and to the loss; explanations inherent to the disappearance and the search; the main changes in life brought on by the loss; their relationships with their community.

The narrations were recorded and transcribed verbatim. The analysis of the text followed the six main phases outlined by Braun and Clarke [30]. Atlas-ti, a computer-assisted qualitative data analysis, was utilised because it permits an examination of the participants’ narratives in terms of principal concepts on the basis of both the prior ideas that were inherent to the research questions and concepts that only became clear as the analysis proceeded [33]. In the following phase, the text was segmented into significant quotations to transform it into hermeneutic unities through the creation of codes (basic categories). Then, the codes were transformed into families (hyper-categories) on the basis of the relationships among the codes. Finally, the relationships between families were sorted according to the conceptualization of anticipatory mourning and ambiguous loss [30,34,35].

Data Sharing and Accessibility: The data that support the findings of this study are available on request from the corresponding author. The data are not publicly available due to privacy or ethical restrictions.

## 3. Results

The qualitative analysis of the interviews showed, in all of the narratives, five prevailing thematic areas according to the DABDA model. As indicated by Kübler-Ross (1969) [16], these did not follow a predetermined sequence. However, the same phases resulted in quite a peculiar form. The following five areas appeared: ‘Incredulity instead of denial’; ‘a centrifuge of anger mixed with shame and resentment towards the community’; ‘bargaining to resolve shame and self-blame’; ‘depression-like mourning without corpse’; and ‘the impossibility of closure: Acceptance of the unknown and non-acceptance of the end’.

### 3.1. Incredulity Instead of Denial

All family members, from the very first moment, were in a state of shock, panic, and confusion because of the disappearance. Elisa, who lost her father and found him dead after nine months after he went missing, said: ‘I lacked air, I really couldn’t breathe. I was desperate...desperate. At night I got all the doubts of what people said. I sometimes got angry because my mother forgot important particulars because of the shock. I thought incessantly if it was possible not to find him at all, if he was alive or dead.’

Similarly, Giuseppe, a retired man whose daughter was never found, said: ‘It is very difficult to manage the panic following the disappearance. I’ve often feared becoming confused.’

Similarly, Alessandra, who had been waiting 26 years for her missing daughter, said: ‘I was shocked and my reaction was to hide myself. Doubts have always macerated me, and even now they torment me that someone has not told me the truth.’ This feeling was also described by Luca, a 61-year-old man whose daughter had disappeared but was finally found alive: ‘It’s like falling into a nightmare and hoping to come out of a really frightening thing. It’s as if you were run over by a running train. It’s everything absurd.’

The incredulity accompanied fear and anxiety, and were linked to the threat of the irreparable. Anna, a 46-year-old woman whose father has never been found, affirmed: ‘He disappeared. But what does disappeared mean? It seems so strange that a person can disappear into the air!’ Incredulity was the cipher of denial that surrounded all of these experiences.

The only way to reduce the uncertainty sprung from the ‘absurdity of annihilation’ was, for all the participants, to conduct an exhausting search for their beloved. Giulia, a 45-year-old teacher whose father was never found, stated: ‘It is impossible that my father was annihilated. This situation is such an absurd thing, he must have gotten lost, he must have been confused, but he’s here and so now we go out and look for him. I assumed that soon someone would call and tell us that they have found him.’ Such a defensive strategy was activated by the relentless desire to embrace the disappeared again. It ran in parallel with the growing anguish as time went by, as Anna explained: ‘I was acting under the action of a mixture of anxiety and desire, hope and hopelessness, confidence in the possibility that others could [go] where I [had not] gone. The moments of inner collapse happened every time I stopped to think and realised that all these feelings and the traces were leading nowhere, but then I started all over again.’ Giuseppe described this condition as: ‘An eternal and insistent waiting for the bell to ring at any moment. But every time I realised the silence and the time that went by, so I prayed to God they hadn’t put my daughter in a bag and thrown her in the trash. I forgave the Lord for taking my daughter away from me, while asking Him to make me sure that she was okay.’ Similarly, Luca said: ‘This silence is absurd and at the same time frightening, because everything is possible and there is no answer. Exploring everything while you [can] see nothing is what insinuates terror.’

### 3.2. A Centrifuge of Anger Mixed with Shame and Resentment towards Community

Anger appears as a consequence of the absurdity of the situation. The conviction of the impossibility that one’s beloved could have become nothing ignites one’s anger, as Giulia described: ‘Not knowing what caused the disappearance and not finding clues to help you reconstruct the events forces you to continue building scenarios of explanation. If you see the disappearance as a car accident, as much as it is a tragedy, you know that the ambulance arrives, the police arrive, the family are notified. Not being able to give a shape to this falling into nothingness makes everything incomprehensible, and every time you have to change your hypothesis the dismay is accompanied by a deep anger.’ The anger burns like a fire, especially when the family members realise that the community does not support them, as Giuseppe pointed out: ‘No one, I say no one has been concerned about this tragedy. It was enough to give me a phone call to show solidarity. I don’t say they had to help me in the search or give me support, but to at least ask me how the search was going. No one. Nobody cared about this absurd disappearance.’

Giulia defined the community’s reaction as: ‘Not only did no one help me get out of this centrifuge of feelings, as I went from one emotion to another with a strong sense of helplessness, but I was also left alone in managing all the formal aspects that the disappearance of a person entails. People look at you as if you were responsible for something. It’s as if they put a mark on you.’ Maria, a 69-year-old woman whose daughter was found dead, said: ‘If you are alone you can’t do anything. Anger is inevitable. Then the association helped me and if it hadn’t, I wouldn’t have been able to find my daughter’s body. Then I could finally mourn her.’ However, anger was also characterised by resentment towards the missing person, who is considered responsible for having thrown his family into a no man’s land, as Elisa described: ‘He put me through nine months of hell, and while I was looking for him my oscillation between hope and despair was interspersed with anger towards him. But my love for him was so strong that I resisted and if I had not found him, now I would still be looking for him.’ Anger was also a reaction to impotence, as Alessandra said: ‘I feel anger, resentment, and disappointment. My life has [been] spent inconclusively looking for someone I have not yet found and I know that it will continue in the search without success, but I cannot give up this hope. This has been my life.’

### 3.3. Bargaining to Resolve Shame and Self-Blame

The participants developed strategies to solve the problem on their own because the community did not offer any valid support. This caused their thoughts to be transformed into continuous ruminations that focused on the desire to return to the past, so as not to make the mistakes that caused the tragedy.

On this basis, recriminations developed from a substrate of guilt, as Anna described: ‘From the first moment I never stopped wondering if I was responsible for all this, and that is why I have always tried to do everything possible to find him. Like I could go back and avoid making the same mistakes. The question was what I could or should have done before to avoid all this. However, this quest for atonement for past faults [was] not always clear. My family and I spent months, years looking for clues along the streets, in every possible city, scrupulously checking every signal. In this way we burned our lives. Everything we were and had before was slowly corroded by this exhausting search under the weight of the feeling of guilt mixed with resentment.’

Alessandra described her thoughts in a similar way: ‘I feel guilty, because even though I have a family of my own, the search and waiting for my missing daughter comes first. I live in resentment towards the community because it corrodes my doubt that someone has not told the truth. But I don’t know if I will ever be able to have the truth about my daughter’s fate because I believe that God doesn’t want to give her back to me, because if she came back I would definitely live only for her and I wouldn’t do anything for the others. I always pray to God to let me die when and how He wants. I tell him that He can take my life back, but in return he must let me know something about my daughter.’ Giulia continued in this direction: ‘Unfortunately, the disappearance of a loved one can divide families that are socially isolated, and so the sense of guilt explodes in recriminations and reprimands of responsibility and blame that trigger angry quarrels and uncontrollable mechanisms. The latent hope is that finding the culprit will also allow us to understand what happened, and therefore to find the missing person. However, these things alienate rather than bring people closer together.’

### 3.4. Depression-Like Mourning without Corpse

The ambiguous mourning was consciously lived and described as a condition of suspension, as Giuseppe said: ‘The whole family lived [in] mourning, even though we never found a corpse. But at the same time, not having had a funeral always reminds us that anything could still happen and all this makes the pain unceasing.’ Maria expressed similar thoughts: ‘The pain is so strong that it doesn’t even give you the strength to react. I felt like I was dead... dead. Without my daughter, my life seemed to be over.’ Giulia described more or less the same emotional experience: ‘I was going on like an automaton. Everything about my father didn’t interest me, it just slipped right through me.’

Alessandra said: ‘Not knowing whether she is alive or dead prevents me from [having closure]. I keep thinking about the last time she said goodbye to me: “Bye mom, see you tonight”, and I’m still there. My life stopped that night when I started to worry. Those who find the body of their missing loved one can at least mourn him on his grave. I can’t do that either.’

Depression occurred within communities that isolated the families in which a person has disappeared, as Elisa said: ‘When a person disappears at first it creates great interest, and it seems that relatives and neighbours want to make themselves useful to find the missing person. Then after a few days no one comes forward. Everyone is afraid of having to continue the search and doesn’t feel like it. So, you stay alone and the others avoid you and leave you in your darkness. Yes, because it’s a dark place, you don’t know where to go, you just go on a grope. It’s very depressive.’

### 3.5. The Impossibility of Closure: Acceptance of the Unknown and Non-Acceptance of the End

Accepting the reality of loss was an extremely complex task for everyone, a goal that, for those who have never heard from their loved one again, has never ended. As Anna explained: ‘It is difficult to put a firm point [on it]. If he died in the mountains, I have to get over it. But the hope is always that he will come back or that we will find him and know what happened. So much time has passed, maybe he is in a dimension where he is better off than here. This is my illusion, which allows me to survive this unconfirmed loss. I think of him as someone who is well.’

Acceptance of the situation was expressed on one side as tolerance towards the unknown, an enveloping darkness in which the disappeared is immersed and where they continue to inhabit the life of the family. Giuseppe said: ‘Martina is always there. Martina is always there even if she is in the dark and I don’t see her. Even when I work and have to think about something else, Martina is with me and my family.’

On the other hand, a dimension that allows us to accept the loss is reality, as Giulia explained: ‘The problems for those who have to manage the disappearance of a family member are not only psychological. I am referring, for example, to retirement, to properties that cannot be sold until after ten years, once the presumed death has been recognised. I am also referring to employers who do not know whether to proceed with the dismissal or not. If you then add all this to the additional problems of everyday life, such as the illness of another family member, you have to come back with your feet on the ground. When my mother got sick, it was a wake-up call for me and I knew that I had to become concrete.’ The ‘concreteness’ of finding the corpse was underlined by Elisa: ‘It was a trauma experienced for the second time, but [it was] different from the first time. When we suddenly found him again, the whole story of waiting and searching became unreal, as if it had been a nightmare that dissolved upon awakening. A tragic awakening, but one that brought me back to reality. An unreal nightmare had ended. I consider myself lucky compared to other people who are still waiting.’

Not all families welcomed the concreteness of death, however. Paola, a 53-year-old policewoman and aunt of a boy who disappeared and was found dead at the age of 24, explained how the parents and the sister of the disappeared coped with their loss:

*I have always been very close to my sister-in-law’s family, but on this occasion, I have tried in every way to help them to overcome their grief. They have not overcome the mourning; they continue to suffer and to not talk about what happened. In my opinion, they still live in expectation even if they know that Giampiero is dead. They didn’t want to see the corpse when he was finally found. In this way it is as if they have chosen to continue as before, as if they were still waiting for him. In fact, they don’t want to talk about this experience, and when someone speaks to them about Giampiero, they suffer profoundly. I would like to help them to solve the problem by talking about it and facing the reality, but I don’t know if it is better to let things go like this. … They preferred to stay in the condition of uncertainty that had accompanied them before he was found and to which they had perhaps become accustomed, because anxiety could be dampened by hope. Giampiero wasn’t there, but he could have been somewhere. When hope disappeared their habit of suspension remained. That’s why talking about Giampiero is painful for them. Because it forces them to talk about the reality of the facts.*

Alternatively, Maria described her font of resilience: ‘For my daughter I did everything I could, even if I didn’t get what I wanted. Now, when I do something for others, I tell you the honest truth, I always seem to do something for her. Kind of like a kind of atonement. That’s why I collaborate with the association.’ Luca, who could embrace his daughter alive, said: ‘When we found her again all the effort, the anguish and despair disappeared. The anger disappeared, there was no more resentment. The relationship has improved, it has become more positive. We have learned to be together in a better way by committing ourselves all to go further, without recriminations.’

## 4. Discussion

The narratives of all participants highlighted the five phases of the DABDA model of coping with grief in a special way. The five areas of thematic relevance that appeared in the narratives were the following: incredulity instead of denial; a centrifuge of anger mixed with shame and resentment towards community; bargaining to resolve shame and self-blame; depression-like mourning without a corpse; and the impossibility of closure: acceptance of the unknown and non-acceptance of the end. It became clear that, in cases of ambiguous loss, each phase of Kubler-Ross’s model must be defined in a different way, while maintaining some key features. Furthermore, many aspects of each narrative could have been classified into more than one theme, which means that the thematic boundaries are permeable, serving mainly as heuristics.

There are, in fact, three particular scenarios that differentiated this experience. The first scenario consisted of those who have not found the loved one. The second consisted of those who found their beloved dead, and the third consisted of those who found their loved one alive. The specificity of those who no longer had news confirmed that they are in a constant condition of anticipatory mourning of an ambiguous type. The lack of a funeral ceremony qualifies this experience as a loss without leave [8]. The silence that characterised the absence appears as a void in which doubts, hopes, expectations, searches, and disappointments were constipated, trapping the griever in ruminative thoughts due to the multiplicity of possible scenarios. Rumination kept the families focused on rethinking the causes and circumstances that led to the disappearance. It also amplified the period of mourning, which manifested in flashbacks that evoke the last exchanges of jokes. Their grief management is arranged as a loss-oriented coping [36,37]. This is characterised by a constant questioning of the reasons for what happened, responsibilities, what could still happen and, mainly, the missing person’s state of life.

The comparison between the narrations of this first group and those of the second allowed us to confirm that, when the corpse is found, this condition of unreality (described as a nightmare) has been overcome. The second trauma, i.e., when there are no longer fantasies regarding discovery, leads to a cessation of the search of the missing beloved. On the contrary, grieving without a corpse to mourn reinforces the disbelief over the loss. This condition prevents the establishment of a process of elaboration of loss through a language of mourning characterised by rituals and symbols that give meaning to the event. This allows for a progressive acceptance of the loss [38]. By celebrating the life of the missing person, the bereaved can consolidate their memories in the present and acknowledge the ambiguity of their mourning within a supportive space reserved for these families. Wayland [26] observed that celebrating the missing person lets the family members feel that the lost person is not resigned to being definitely deceased or gone forever; it allows them to celebrate the beloved one as a person instead of as ‘the one who disappeared’, an effect confirmed by Giulia. Indeed, Giulia’s whole narrative seems to indicate that finding the corpse permits the family to stop their allegations, which continue to oscillate until the fate of the missing person is known. To better describe the form of mourning triggered by ambiguous loss, Wayland [14], following the studies of Boss [6], utilised the dual process model, which refers to an oscillation between mourning the potential death of the beloved and maintaining hope that the beloved may still be alive [6,14,26,36]. We can also recognise this form of oscillation in Alessandra’s narrative, especially in the final theme. However, Paola’s narrative clearly shows that hopeless waiting may be the preferred solution, freezing the oscillation and the work of mourning rather than confronting reality.

Moreover, it turns out that the community is unable to sustain this kind of uncertainty for a long time. It is also unable to support the families living this experience, because it is perceived as destabilizing [4,9]. In fact, the closeness of the community is generally felt only in the first weeks after the disappearance. After this period, the community moves away and isolates the family, which withdraws in a deep sense of shame with experiences of stigmatization and loneliness. The absence of the community binds mourners to the conviction that they will be able to solve the problem on their own at all costs, leading them to constantly undertake new research strategies. We find in this profile something similar to what is described as disenfranchised grief, i.e., those situations in which society does not know how to recognise the suffering of the sorrowful because it cannot give meaning to particular forms of loss [39,40]. In these cases, the community becomes a kind of limbo in which all relationships are characterised by ambiguity.

This type of experience can lead to the stigmatization of grieving families, as their grief management practices do not align with people’s expectations about loss, and this, in turn, can lead to additional stress amongst family members as their loss is not socially acknowledged [41,42]. Because the phenomenon of ambiguous loss is still poorly understood and susceptible to clichés, families do not often share their emotions with others for fear that they will misunderstand these feelings or minimise the situation. Professionals who support such families must make the therapy space a safe place, like a ‘fence’ that protects against misunderstanding, but they must also work with families to restore relations with the community, as this can help them to face their situation [26].

A similar type of grief is the traumatic mourning experienced by survivors of a loved one’s suicide. These two types of grief share the presence of experiences like guilt, resentment, and anger. In addition, there is a presence of incessant ruminations related to the search for explanations, as well as feelings of shame and the perception of social stigmatization [43]. However, when the lifeless body is found, this corresponds to a further shock for the family because all hopes of finding it are eclipsed. However, the path of complete mourning can begin, as without evidence of death there is no resolution of loss. The work of acceptance within families centers on the condition of ambiguity: the aim of therapeutic intervention is to help families find ways to develop tolerance for ambiguity so that their relationship with the beloved is not wholly characterised by this traumatic experience [44]. At the same time, professionals must remember that dealing with death in a definitive way can be unsustainable for some patients. As Paola indicated, even when there is the possibility of closure, the family may decide to remain suspended by refusing to witness the concreteness of the beloved’s corpse. Giampiero’s family has clung to a psychological condition similar to waiting, but without hope.

Adequate psychological help could aid in the process of reconstructing the meaning of the loss, which Neimeyer [45] described as ‘sense-making.’ This process, together with a focus on the needs of the family instead of the details of the investigation, helps families to ascribe meaning to their loss. In terms of psychological support, it is also necessary to give families a space in which to celebrate their loved ones. This can be achieved in various ways; the therapist’s task is to help the family find the most significant and suitable route for their circumstances. These celebrations can generate creative connections between loved ones and the missing person, celebrating them not to remember their loss, but to honour their life and recognise that they are not present for now [26]. ‘Benefit-finding’ is the finding of something positive in the lived experience. In both aspects, a community factor that seems to help even those who no longer have news of their loved one is linked to the possibility of allowing them to dedicate themselves to those who live the same experience. In this study, the participants collaborated with an association dedicated to such cases. The resilience that these initiatives activate is the result of the ability to transform uncertainty into an inner strength that gives relief to other people who have to face a similar path [46]. Solidarity becomes the new life project that gives meaning to a disappearance, as well as a new way to feel close to the lost person. The desire to help other families living with the pain of disappearance activates a need to cope by finding a resolution [47]. However, many families find it difficult to perceive this unique loss as an opportunity. It is also worthwhile to help families acknowledge the steps they have taken, or the progress achieved during the person’s disappearance, because often they are not aware of this and see no changes. This may also help family members to envision a path together, whereby they may survive their ambiguous loss. Before having family members share their own experiences, professionals may opt to share the experiences of other families to highlight how they reacted to the same situation, thereby creating a context or distributed community to ground their pain [26].

Despite Boss’s [7] assertion that mourning is an oscillating process of ups and downs [48], more so than a series of grief stages (as per Elisabeth Kübler-Ross [16]) or a construct of anticipatory mourning (as per Lindemann [15]), our study suggests that ambiguous loss and anticipatory mourning share many qualities, and that research on each of these topics can improve our comprehension of loss without closure.

## 5. Conclusions

Our study outlines the need to set up specific interventions aimed at supporting relatives who find themselves having to deal with the disappearance of a family member. This is a highly destabilizing event with the characteristics of anticipatory mourning, which then turns into prolonged and complicated mourning due to the uncertainty that blocks the processing of the loss. From the moment of the disappearance, different scenarios may appear: that the missing person will be found alive; that the relative will receive news of their loved one’s death; and that there will be no further news of their loved one. The last situation freezes the experience of loss for the relative of the missing person, triggering an endless exercise in searching for and hoping to find the missing person. The endless waiting for the return of the beloved one and the lack of a corpse does not allow the transition from anticipatory grief to complete mourning. This results in an ambiguous grieving.

The complex nature of this undefined loss therefore highlights the need to have an in-depth understanding of this experience. Any support for these grievers must take into account the ambivalent condition of this event. Difficulties in adapting to the loss must therefore be supported by appropriate community and psychological interventions that make it possible to work out the acceptance of a new condition marked by uncertainty. Through adequate psychological support the event of disappearance, as is the case with other traumatic experiences, could then become the starting point for a personal transformation of the individual. This is known as post-traumatic growth, understood as a path of positive change for an individual following trauma.

Further investigations will make it possible to recognise more deeply the relationship between different traits of this mourning without closure and its oscillations. It might therefore be useful to better consider its specific expressions of the dual process model, describing how and which kind of loss-oriented stressors interact with the restoration-oriented process. Indeed, it could be useful to specifically describe the relationships between the feelings related to the loss and the tasks that mourners are convinced they have to perform to change the situation and to overcome the suffering. Thus, it would be recognisable functional and dysfunctional psychological strategies that characterize this specific form of loss and its mourning.

## 6. Limits and Future Studies

The main limitations of the study are (1) the small number of participants and (2) the unsystematic treatment of the differences between modes of being ‘missing’ (survivors, deceased, still missing). The small number of participants prevents the recognition of sufficiently stable narrative subgroups to describe well-structured, differentiated profiles. Future studies should not only outline these profiles, but also consider the relative effects that television search programmes produce in families. The findings of the present research cannot be generalised; however, they provide a much-needed foundation for future research on missing persons in Italy, thereby filling a gap in the literature. In fact, further studies should better investigate aspects of this kind of loss, such as specific defensive psychological strategies and forms of attribution of responsibility in relation to depression. Furthermore, it could be very useful to analyse the nature of the perceived discrimination, as well as the effects of the media collaboration.

## Figures and Tables

**Table 1:** Degree of relationship with the missing person.

Relationship	Survivors	Deceased	Missing
Parent	1	1	2
Son/daughter	-	1	2
Aunt	-	1	-
Total	1	3	4
